# Digital clubbing in tuberculosis – relationship to HIV infection, extent of disease and hypoalbuminemia

**DOI:** 10.1186/1471-2334-6-45

**Published:** 2006-03-10

**Authors:** Henry Ddungu, John L Johnson, Marek Smieja, Harriet Mayanja-Kizza

**Affiliations:** 1 Department of Medicine, Makerere University and Mulago Hospital, Kampala, Uganda; 2 Department of Medicine, Division of Infectious Diseases, Case Western Reserve University and University Hospitals of Cleveland, Cleveland, Ohio, USA; 3 Departments of Pathology and Molecular Medicine, Medicine, and Clinical Epidemiology and Biostatistics, McMaster University, Hamilton ON, Canada; 4 L424 St. Joseph's Hospital, 50 Charlton Ave. E., Hamilton ON L8N 4A6, Canada

## Abstract

**Background:**

Digital clubbing is a sign of chest disease known since the time of Hippocrates. Its association with tuberculosis (TB) has not been well studied, particularly in Africa where TB is common. The prevalence of clubbing in patients with pulmonary TB and its association with Human Immunodeficiency Virus (HIV), severity of disease, and nutritional status was assessed.

**Methods:**

A cross-sectional study was carried out among patients with smear-positive TB recruited consecutively from the medical and TB wards and outpatient clinics at a public hospital in Uganda. The presence of clubbing was assessed by clinical signs and measurement of the ratio of the distal and inter-phalangeal diameters (DPD/IPD) of both index fingers. Clubbing was defined as a ratio > 1.0. Chest radiograph, serum albumin and HIV testing were done.

**Results:**

Two hundred patients (82% HIV-infected) participated; 34% had clubbing by clinical criteria whilst 30% had clubbing based on DPD/IPD ratio. Smear grade, extensive or cavitary disease, early versus late HIV disease, and hypoalbuminemia were not associated with clubbing. Clubbing was more common among patients with a lower Karnofsky performance scale score or with prior TB.

**Conclusion:**

Clubbing occurs in up to one-third of Ugandan patients with pulmonary TB. Clubbing was not associated with stage of HIV infection, extensive disease or hypoalbuminemia.

## Background

Digital clubbing is a well described finding in various pulmonary, cardiovascular, neoplastic, infectious, hepatobiliary and gastrointestinal diseases[1]. However, tuberculosis (TB) is not listed in the differential diagnosis of clubbing in some major modern textbooks of medicine[2]. In an earlier study in Nigeria, MacFarlane et al reported clubbing in 21% of patients with pulmonary TB and noted associations with severity of disease, cavitary TB and Hypoalbuminemia[3].

Few recent studies and none to our knowledge during the human immunodeficiency virus (HIV) pandemic have examined the prevalence of finger clubbing in patients with pulmonary TB and its relationship to HIV co-infection, severity and chronicity of TB disease, and nutritional status. TB is a major public health problem in sub-Saharan Africa with a case rate exceeding 377 per 100,000 population per year in Uganda[4]. Forty thousand six hundred and ninety-five new TB cases (19,088 smear-positive) were registered with the Uganda National TB and Leprosy Programme (NTLP) in 2002[4]. We performed a cross sectional study of the prevalence of digital clubbing and its clinical correlates in consecutive adults with pulmonary TB presenting to Mulago Hospital, the largest public teaching hospital in Uganda.

## Methods

### Study population

All adult patients with sputum acid-fast bacilli (AFB) smear positive pulmonary TB presenting to Mulago Hospital between December 2002 and March 2003 were eligible for study participation. Patients were ineligible if they had extra-pulmonary TB with negative sputum smears, sputum smear negative pulmonary disease or diseases including empyema, cyanotic congenital heart diseases, bronchogenic carcinoma, and ulcerative colitis, all known to be associated with clubbing. The study protocol was reviewed and approved by the Research and Ethics Committee of Makerere University Medical School. All subjects gave informed consent for study participation and HIV testing and received pre- and post-HIV-test counseling. HIV seropositive individuals were further categorized using the World Health Organization (WHO) Clinical Staging System for HIV Infection and Disease[5].

We examined 115 general medical patients (irrespective of diagnosis), at the Mulago emergency medical ward, and 143 healthy volunteers for presence of finger clubbing, in order to estimate the prevalence ofclubbing in these populations. No further follow-up studies were carried out on these individuals.

### Measurements

All patients with TB underwent medical history and physical examination including determination of their Karnofsky performance scale score[6]. The Karnofsky scale is a well-validated assessment of an individual's health and well-being based on a performance index of physical ability. It is often used in clinical research including HIV research to monitor and record the health of patients. The Karnofsky score uses a scale from 0 to 100, where 100 is when the patient has no complaints or evidence of disease and 50 is when the patient requires considerable assistance and frequent medical care.

Clubbing was assessed by clinical signs and by determination of the phalangeal depth ratio. The fingers were examined for presence of increased nail curvature, nail bed fluctuation, Schamroth's sign[7] (where opposing of fingers of both hands leads to disappearance of the normal 'diamond' window that forms between the nail beds) and definite clubbing and the findings recorded. For purposes of this study, definite clubbing was defined as finger clubbing that was obvious to the examiner's eyes without need for any measuring tools. Then, using vernier calipers (Stanley Tools, New Britain, CT), the distal phalangeal (DPD) and interphalangeal (IPD) diameters of both the right and left index fingers were measured and the phalangeal depth (DPD/IPD) ratio was calculated [8]. The greater of the DPD/IPD ratios for the index fingers was used in determining the presence or absence of clubbing. Clubbing was considered present if the DPD/IPD ratio was greater than 1.0 [8]. The DPD/IPD is a well-validated objective measure of the presence of clubbing with acceptable reproducibility. The mean normal DPD/IPD ratio is approximately 0.895 and is independent of age, race and sex[9,10]. A cut-off point of greater than 1.0 exceeds the normal range by about 2.5 standard deviations. In this study, the DPD/IPD ratio was regarded as the best (gold standard) test for clubbing for use in comparison with other findings.

Sputum AFB smears were performed by the Ziehl-Neelsen method and graded according to IUATLD guidelines[11]. A standard postero-anterior chest radiograph was obtained for each patient. The radiographic severity of disease was graded as minimal, moderately advanced, or far advanced disease using the United States National TB and Respiratory Disease scheme[12]. The number of lung zones (out of 6) and the presence of specific findings also were recorded. A cavity was defined as a gas-containing lucent space at least 1 cm in diameter within the lung parenchyma surrounded by an infiltrate or fibrotic wall greater than 1 mm thick. HIV serostatus was determined by HIV1/2 enzyme immunoassay using Determine™ HIV 1/2 kit (Abbott Laboratories, Tokyo, Japan). Serum albumin was measured by using the ALBUMIN Bromocresol Green™ kit (Randox Laboratories Ltd, Diamond Road, Crumlin, UK); the normal range being 3.8 to 4.4 g/dL.

History and physical examination and assessment of clubbing were performed prior to HIV testing. Laboratory technologists performing sputum AFB smears, HIV and albumin determinations and radiologists reviewing the chest radiographs were masked to all clinical data and the presence of clubbing.

### Statistical analysis

Estimation of the sample size was based on the estimated prevalence of finger clubbing of 25% and a precision of 5%. Data were analyzed using SPSS version 10.0 (SPSS, Inc., Chicago, IL). Tests of significance using p-values, Odds ratios and Likelihood ratios, were made using 2 by 2 tables. Confidence intervals and likelihood ratios were calculated using Confidence Interval Analysis (CIA) for windows version 2.1.1. Because of our small sample size and the small number of cases in some groups, we did not perform a multivariable analysis.

For statistical purposes patients were put into two HIV clinical categories. Those in WHO clinical stages 1 and 2 were categorized as early HIV disease whereas those in stages 3 and 4 were classified as late disease. Karnofsky performance scale scores were analyzed by dichotomizing at 70; those with performance scores equal to or greater than 70, where 70 is defined as being able to care for self, but unable to carry on normal activity or to do active work, and those with scores less than 70. A level of significance of p < 0.05 was used to reject the null hypothesis in all analyses.

## Results

### Study population

During the study period 200 patients with smear positive pulmonary TB were approached. All consented to participate in the study. Fifty-nine percent were males. The mean age ± SD was 32.6 ± 11.3 years. The most frequent symptoms reported by the patients were cough for more than three weeks (99%), sputum production (95%), fever (95%), night sweats (90%) and weight loss (86%). Twenty-four percent reported hemoptysis. Twelve percent had received prior TB treatment within the previous year and 82% were HIV-infected. Of the patients with HIV infection, 58% had early HIV infection and 42% had late stage disease.

We also evaluated 258 adult subjects including 115 newly admitted general medical patients (57% males; mean age 36 years) and 143 healthy medical students and hospital staff volunteers (48% males; mean age 22 years) for the presence of clubbing.

### Prevalence of clubbing in patients with TB and control subjects

Using a DPD/IPD ratio of greater than 1.0 as the gold standard, we found that 60 (30%) of 200 patients with pulmonary TB had digital clubbing. Twenty-one (18%) of 115 general medical patients and 3 of 143 (2%) healthy volunteers had clubbing.

The clinical signs of finger clubbing identified were nail bed fluctuation, increased nail curvature, a positive Schamroth's sign and definite clubbing and were distributed as 29.0%, 26.5%, 20.5 and 18.5% respectively, among the study participants. Twenty-three patients (11.5%) had only one of these clinical signs, 18 (9.0%) two clinical signs, 14 (7.0%) while 22 (11.0%) had four clinical signs. One hundred twenty three patients (61.5%) did not have any sign of clubbing.

We compared the proportion of subjects determined to have clubbing diagnosed by measurement of the DPD/IPD ratio with other clinical methods and signs (Table 1). The two methods differed significantly as shown in table 1. Presence of nail bed fluctuation, increased nail curvature, a positive Schamroth's sign and obvious clubbing were significantly associated with finger clubbing as per the gold standard. Having all four clinical signs was highly associated with clubbing (p < 0.001; likelihood ratio 49.0; 95% CI 6.7 to 356), whereas having none of the clinical signs was significantly associated with absence of clubbing (p < 0.001; likelihood ratio 0.3; 95% CI: 0.2 to 0.5). Presence of only one clinical sign and two clinical signs had a very low sensitivity for clubbing (Table 1). Compared with the other clinical signs, nail-bed fluctuation had a higher sensitivity of 72% (95% CI: 59.2% to 81.5%) and a good specificity of 89.3% (95% CI: 83.1% to 93.4%). The proportion of patients with pulmonary TB who had clubbing did not differ by HIV serostatus. Fifty of 163 (31%) HIV-seropositive patients had clubbing as compared to 10 out of 37 (27%) of those who were HIV-seronegative (Odds ratio 1.2; 95% CI: 0.5 to 2.9; p = 0.66). Presence of clubbing was not associated with age, sex, or severe symptoms of TB such as hemoptysis (data not shown). Twenty-one percent of patients with performance status of 70% and above (23 of 108), had clubbing compared with 40% (37 of 92), of those who had lower performance capacity. Patients with a performance status score below 70% were more likely to have clubbing (OR 2.49, 95% CI: 1.3 to 2.9; p = 0.003) than those with a higher (able to care for self) performance. Clubbing was more frequent in patients who had received prior anti-TB treatment compared to those who had not been treated previously for TB (OR 2.7; 95% CI: 1.0 to 6.9; p = 0.023).

**Table 1:** Clinical signs of clubbing compared with Gold standard measure

**Clinical Sign**	**"Gold standard" measurement**		**Sensitivity ** (%) (95%CI)	**Specificity ** % (95%CI)	**Positive LR ** (95%CI)	**Negative LR ** (95%CI)	**p-*Value***
						
	**Clubbing** ** (n = 60)**	**No Clubbing** ** (n = 140)**					
Nail bed fluctuation (n = 58)	43	15	71.7 (59.2, 81.5)	89.3 (83.1, 93.4%)	6.7 (4.1, 11.1)	0.3 (0.2, 0.5)	< 0.001
Increased nail curvature (n = 53)	38	15	63.3 (50.7, 74.4%)	89.3 (89.3, 93.4%)	5.9 (3.5, 9.9)	0.4 (0.3, 0.6)	< 0.001
Schamroth's sign (n = 41)	30	11	50.0 (37.7, 62.3%)	92.1 (86.5, 95.6%)	6.4 (3.4, 11.8)	0.5 (0.4, 0.7)	< 0.001
Definite clubbing (n = 37)	31	06	51.7 (39.3, 63.8%)	95.7 (91.0, 98.0%)	12.1 (5.3, 27)	0.5 (0.4, 0.7)	< 0.001
At least one sign (n = 76)	47	29	78.3 (66.4, 86.9%)	79.3 (71.8, 85.2%)	3.8 (2.7, 5.4)	0.3 (0.2, 0.4)	< 0.001
Two or more signs (n = 54)	41	13	68.3 (55.8, 78.7%)	90.7 (84.8, 94.5%)	7.4 (4.3, 12.7)	0.3 (0.2, 0.5)	< 0.001
No Clinical Sign (n = 123)	13	110	21.7 (13.1, 33.6%)	21.4 (15.4 – 28.9%)	0.3 (0.2, 0.5)		< 0.001
One Clinical Sign (n = 23)	06	17	10.0 (4.9, 20.1%)	87.9 (81.4, 92.3%)	0.8 (0.3, 2.0)		0.633
Two Clinical Signs (n = 18)	08	10	13.3 (6.9, 24.2%)	92.9 (87.4, 96.1%)	1.9 (0.8, 4.5)		0.130
Three Clinical Signs (n = 14)	12	02	20.0 (11.8, 31.8%)	98.6 (94.9, 99.6%)	14.0 (3.2, 61)		< 0.001
Four Clinical Signs (n = 22)	21	01	35.0 (24.2, 47.6%)	99.3 (96.1, 99.9%)	49.0 (6.7, 356)		< 0.001

LR = Likelihood Ratio; CI = Confidence Interval

Next, we examined whether the radiographic extent of disease and presence of cavitary disease on chest radiograph were associated with the presence of clubbing. The proportion of patients with clubbing did not differ between patients with more lung zones involved by disease or bilateral disease, those with minimal as compared to radiographically moderate advanced or far advanced disease, and those with any visible cavitary disease (Table 2).

**Table 2:** Patient and radiographic characteristics in relationship to clubbing as defined by the phalangeal depth ratio in patients with smear positive pulmonary tuberculosis

**Characteristic**	**"Gold standard" measurement**		**p-Value**	**Odds Ratio (95% Confidence limits)**
			
	**Clubbing (n = 60)**	**No Clubbing (n = 140)**		
Hypoalbuminemia (n = 48)	19	29	0.097	1.8 (0.9 – 3.7)
HIV Seropositive (n = 163)	50	113	0.662	1.2 (0.5 – 2.9)
Karnofsky score ≥ 70% (n = 108)	23	85	0.003	0.40 (0.2 – 0.8)
Karnofsky score less than 70% (n = 92)	37	55	0.003	2.5 (1.3 – 4.9)
Prior TB treatment history (n = 24)	12	12	0.023	2.7 (1.0 – 6.9)
**Extent of disease on Chest Radiograph**				
Minimal Disease (n = 36)	10	26	0.747	0.9 (0.4 – 2.1)
Moderate Disease (n = 98)	28	70	0.666	0.9 (0.5 – 1.7)
Advanced Disease (n = 65)	21	44	0.621	1.2 (0.6 – 2.3)
Bilateral Lung disease (n = 164)	52	112	0.261	1.6 (0.7 – 4.2)
Cavitary Disease (n = 85)	27	58	0.640	1.2 (0.6 – 2.2)

We used serum albumin as a measure of nutritional status to assess the relationship of clubbing and nutrition. Mean serum albumin was 4.0 ± 0.7 g/dL. Seventy-six percent of the patients with TB had a normal serum albumin. Eight percent had levels less than 2.5 g/dL. Forty-eight patients (24%) had serum albumin level below the normal range and, of these, 40% had finger clubbing compared to 27% of those with normal serum albumin levels. Subnormal albumin (less than 2.5 g/dL) was not associated with presence of clubbing (OR 1.8, 95% CI: 0.9 to 3.7; p = 0.097).

## Discussion

In a study of consecutive patients carried out in a large public hospital we found that digital clubbing, as objectively defined by an interphalangeal depth ratio greater than 1.0, was present in nearly one-third of adults with pulmonary TB in sub-Saharan Africa. Nail bed fluctuation, best elicited by gently rocking the nail at the base of the nail bed, was found to be a better quick bedside assessment tool for presence of finger clubbing as compared with other clinical findings.

In their systematic review of the precision and accuracy of clinical examination for clubbing, Myers and Farquhar recommended use of the profile angle and phalangeal depth ratio (measured using calipers) as quantitative indices in identifying clubbing[13]. We used the phalangeal depth ratio in this study because it is more reproducible and easier to perform than measuring the hyponychial (profile) angle.

In our study, HIV co-infection, sputum bacillary load, radiographic extent of TB, cavitary disease and serum albumin, were not associated with the presence of clubbing. Poor performance status and a history of prior TB were associated with finger clubbing.

Our findings of a prevalence of finger clubbing of 30% among 200 sputum smear positive patients with pulmonary TB is similar to a report of 34% in Malawi[14] but higher than the 16 to 19% prevalence reported in other earlier studies from Africa and the Middle East[3,15,16]. These studies were smaller than the current study and two were carried out before the HIV pandemic. Prior TB was significantly associated with clubbing in our study. Residual bronchiectasis is frequent in areas of healed TB and may have contributed, in part, to this finding.

We found no association between HIV infection and clubbing in patients with pulmonary TB. The proportion of patients with TB who had digital clubbing was comparable between HIV-infected and HIV-uninfected patients. We did not assess the frequency of clubbing in HIV-positive patients without TB in our study; however, earlier reports suggest that clubbing is infrequent in adults with HIV/AIDS and, when present, is usually due to another underlying cause[17]. In another study from India, none of 120 asymptomatic HIV-infected adults had clubbing[18]. In that report, six of 90 patients with symptomatic AIDS had clubbing and, of these, four had TB.

We were unable to confirm an earlier report from Nigeria[3] in the pre-HIV era that clubbing was more frequent in patients with hemoptysis or cavitary TB, characteristics suggestive of extensive or chronic TB. Similar to other studies[14,15] we observed no correlation between clubbing and hemoptysis, the extent of disease on chest radiograph, or the presence of cavitary disease. We are unsure whether the high prevalence of HIV infection in our patients contributed towards this lack of association.

Only 2% of the healthy volunteer we examined had clubbing; however, the 18% prevalence of finger clubbing identified among newly admitted general medical patients in our study was higher than expected. The 18% prevalence of clubbing in these patients may have been due to unrecognized TB or other conditions associated with clubbing.

The pathogenesis of clubbing in TB and other diseases is unknown. Many studies have demonstrated increased blood flow in the clubbed portion of the fingers except in hereditary clubbing[19]. The exact mechanism through which increased blood flow results in changes in the vascular connective tissues under the nail beds is also unknown. Leading hypotheses include the presence of a circulating vasodilator[20], derived from the association of clubbing with cyanotic congenital heart disease; a neural mechanism[1] with particular consideration of the vagal system; chronic hypoxia[20] as in congenital cyanotic heart disease; a genetic predisposition[21] and, more recently, a role for platelet derived growth factor (PDGF), a multifunctional cytokine growth factor[22,23]. PDGF is released from fragments of platelet clumps or megakaryocytes. It has been hypothesized that platelet fragments may lodge in the vascular beds of the fingertips where they release PDGF. PDGF has general growth-promoting activity and causes increased capillary permeability and connective tissue hypertrophy, hence clubbing.

Our study has several limitations. We performed a single center study and patients at our hospital may not be representative of all patients with pulmonary TB in Uganda.

However, our sample size (200) was substantial when compared to other African studies, and Mulago Hospital is the largest referral centre and TB clinic in Uganda. Second, we excluded patients with sputum smear negative TB who have a lower bacillary burden. Patients with advanced HIV disease are more likely to be sputum smear negative than patients who are HIV non-infected[24] and our sample may be biased towards patients with earlier HIV disease. We studied patients with sputum smear positive because this is the most commonly used test for TB diagnosis in sub-Saharan Africa. Due to fiscal constraints, we were unable to perform CD4 lymphocyte counts on our patients to categorize their degree of HIV-related immunosuppression; however, the WHO clinical stage of HIV infection, a widely used assessment in the care of HIV-infected adults in resource constrained setting was used as a measure of the stage of HIV infection.

Third, sputum cultures for organisms other than mycobacteria were not done, making it difficult to exclude possible concomitant pyogenic infections leading to suppurative lung disease and bronchiectasis, which could by itself cause finger clubbing. Fourth, due to limited resources, we were unable to perform extensive investigations for specific underlying conditions known to be associated with clubbing. It is therefore difficult to attribute the 30% prevalence of clubbing to pulmonary TB alone. However, comprehensive efforts were undertaken to exclude all patients presenting with a suggestive history of such conditions. We lacked a multivariable analysis approach hence making it not possible to determine which of the factors were independently associated with clubbing

Our assessment of nutritional status in the study was limited to body mass index (BMI) and serum albumin though other measurements such as skin fold measurements might have provided more comprehensive information about nutritional status. Lastly, the presence of congenital clubbing, which is indistinguishable from acquired clubbing[1], could not be excluded. Congenital clubbing is infrequent; however, and unlikely to affect our results.

## Conclusion

In conclusion, our data show that clubbing is a frequent clinical finding in patients with pulmonary TB in sub-Saharan Africa and supports this diagnosis. Its presence appears to be unaffected by concomitant HIV disease, the radiographic extent of disease and nutritional status, as assessed by hypoalbuminemia. Our findings could be applied to the routine clinical care of patients by having clinicians look out for presence of finger clubbing. Trained physicians can reliably and consistently assess the presence of clubbing[25]. As noted by Graham et al, in areas where medical resources are scarce, the presence of clinical findings that can improve diagnostic performance may be highly useful[26].

## Competing interests

The author(s) declare that they have no competing interests.

## Authors' contributions

HD, JJ and HMK were involved in the design and execution of the study. HD, JJ and MS contributed to the analysis, and all authors contributed to manuscript writing.

## Pre-publication history

The pre-publication history for this paper can be accessed here:


